# Use of bacteria for improving the lignocellulose biorefinery process: importance of pre-erosion

**DOI:** 10.1186/s13068-018-1146-4

**Published:** 2018-05-23

**Authors:** Shengnan Zhuo, Xu Yan, Dan Liu, Mengying Si, Kejing Zhang, Mingren Liu, Bing Peng, Yan Shi

**Affiliations:** 1 School of Metallurgy and Environment, Central South University, Changsha, 410083 China; 2 Chinese National Engineering Research Center for Control & Treatment of Heavy Metal Pollution, Changsha, 410083 China

**Keywords:** Bacterial pretreatment, Pre-erosion, *Pandoraea* sp. B-6, Extracellular enzymes, Lignin, Mediator, Laccase, Manganese peroxidase

## Abstract

**Background:**

Biological pretreatment is an important alternative strategy for biorefining lignocellulose and has attracted increasing attention in recent years. However, current designs for this pretreatment mainly focus on using various white rot fungi, overlooking the bacteria. To the best of our knowledge, for the first time, we evaluated the potential contribution of bacteria to lignocellulose pretreatment, with and without a physicochemical process, based on the bacterial strain *Pandoraea* sp. B-6 (hereafter B-6) that was isolated from erosive bamboo slips. Moreover, the mechanism of the improvement of reducing sugar yield by bacteria was elucidated via analyses of the physicochemical changes of corn stover (CS) before and after pretreatment.

**Results:**

The digestibility of CS pretreated with B-6 was equivalent to that of untreated CS. The recalcitrant CS surface provided fewer mediators for contact with the extracellular enzymes of B-6. A pre-erosion strategy using a tetrahydrofuran–water co-solvent system was shown to destroy the recalcitrant CS surface. The optimal condition for pre-erosion showed a 6.5-fold increase in enzymatic digestibility compared with untreated CS. The pre-erosion of CS can expose more phenolic compounds that were chelated to oxidized Mn^3+^ and also provided mediators for combination with laccase, which was attributable to B-6 pretreatment. B-6 pretreatment following pre-erosion exhibited a sugar yield that was 91.2 mg/g greater than that of pre-erosion alone and 7.5-fold higher than that of untreated CS. This pre-erosion application was able to destroy the recalcitrant CS surface, thus leading to a rough and porous architecture that better facilitated the diffusion and transport of lignin derivatives. This enhanced the ability of laccase and manganese peroxidase secreted by B-6 to improve the efficiency of this biological pretreatment.

**Conclusion:**

Bacteria were not found useful alone as a biological pretreatment, but they significantly improved enzymatic digestion after lignocellulose breakdown via other physicochemical methods. Nonetheless, phenyl or phenoxy radicals were used by laccase and manganese peroxidase in B-6 for lignin attack or lignin depolymerization. These particular mediators released from the recalcitrance network of lignocellulose openings are important for the efficacy of this bacterial pretreatment. Our findings thus offer a novel perspective on the effective design of biological pretreatment methods for lignocellulose.

**Electronic supplementary material:**

The online version of this article (10.1186/s13068-018-1146-4) contains supplementary material, which is available to authorized users.

## Background

Current techniques to pretreat biomass include chemical, physical, physicochemical, and biological pretreatments [1, 2]. Among them, biological pretreatment appears to be a promising technique with no harsh chemical requirements, a low energy input, mild operational condition, reduced capital costs, and low formation of inhibitors [3–5].

Over the past few decades, the fungal pretreatment of biomass has been widely investigated and applied, because of the unique advantage that fungi can secrete ligninolytic enzymes to degrade lignin and enhance the efficiency of enzymatic hydrolysis [4, 6]. For example, white rot fungus such as *Phanerochaete chrysosporium*, *Ceriporiopsis subvermispora*, *Echinodontium taxodii*, *Physisporinus vitreus*, *Pleurotus*, and *Bjerkandera* species [7–9] is able to produce ligninolytic enzymes, including manganese peroxidase (MnP), lignin peroxidase (LiP), versatile peroxidase (VP), and laccase (Lac). These enzymes oxidize the lignin polymer and generate aromatic radicals [10, 11], after which C4-ether breakdown, aromatic ring cleavage, C_*α*_–C_*β*_ breakdown, and demethoxylation occur. Thus, lignin depolymerization is achieved [11]. To obtain higher sugar yield and pretreatment by-products (e.g., furfural, 5-hydroxymethylfurfural and levulinic acid) [12], combining fungal pretreatment with other pretreatment methods such as acid pretreatment [13] and steam explosion [14], is gradually being developed [15]. Interestingly, the efficiency of enzymatic hydrolysis is better than fungal pretreatment or other pretreatment alone, whether the fungal pretreated step was set before or after other pretreatments [13, 14, 16–23]. However, fungal growth is time consuming, and the delignification rates of lignin are low [4, 6, 14, 24]. Thus, normally biological pretreatment is not the industrial reality due to the low reaction rate and the long-time processes [25]. Moreover, although an individual fungus (e.g., *Phanerochaete chrysosporium*) can grow relatively fast, it lacks the ability to selectively degrade lignin and holocellulose [7, 14]. It also provides hydrolytic enzymes during its growth process that break down carbohydrates, resulting in the loss of cellulose and decreased sugar yield [7]. Additionally, the brown rot fungus (e.g., *Gloeophyllum trabeum*) plays a role in modifying lignin to remove hemicellulose and cellulose with its ability to circumvent the lignin barrier [26].

Compared with fungi, then, although many bacteria like *Novosphingobium* sp. B-7, *Cupriavidus basilensis* B-8, and *Comamonas* sp. B-9 have been screened for lignin degradation [27–29], reports remain scarce on the bacterial pretreatment applied to biomass directly, undermining our understanding of the mechanism underpinning the bacterial pretreatment of biomass. The bacterial degradation of lignin is divided into two steps: depolymerization of extracellular lignin and degradation of intracellular lignin-derived aromatic compounds [30]. Some bacterial extracellular peroxidases capable of significant lignin degradation activity have been reported, such as LiP [31], Dye-decolorizing peroxidase [32], Lac [33, 34], and MnP [35]. In our previous research, we found that *Pandoraea* sp. B-6 (hereafter, B-6), which was isolated from erosive bamboo slips, could produce two extracellular enzymes (i.e., MnP and Lac) with high activity in the process of degrading Kraft lignin and lignin derivatives [36]. Of these two kinds of extracellular enzymes, Lac is a multi-copper oxidase that oxidizes a broad range of substrates, such as various substituted phenolic compounds [34, 37]. Lignin-derived compounds, including phenolic aldehydes, ketones, acids, and esters related to the three lignin units including syringyl (S), guaiacyl (G), and *p*-hydroxyphenyl (H), are efficient Lac mediators for lignin degradation [38]. The MnP can modify lignin and has the capability of oxidizing and depolymerizing both natural and synthetic lignins [39]. MnP produced by B-6 is inferred to be a novel enzyme or isozyme [27]. Furthermore, we discovered that B-6 has enzymes related to H_2_O_2_ generation (e.g., aryl alcohol oxidase, glucose oxidase, cellobiose dehydrogenase, pyranose-2-oxidase). These oxidases can assist the oxidation of Mn^2+^ to Mn^3+^, after which the chelated Mn^3+^ acts as a diffusible redox mediator that attacks the phenolic lignin structures to phenoxy radicals [39]. In addition, B-6 cannot use various sugars as carbon sources including glucose, galactose, xylose, arabinose, and mannose (unpublished data). Based on these, it is reasonable to hypothesize that bacterium B-6 could use their extracellular lignin-degrading enzymes to deconstruct the lignin exclusively in a biomass pretreatment. In a recent work, we found that bacterial strain *Cupriavidus basilensis* B-8 (hereafter, B-8) was able to enhance the enzymatic digestibility highly after dilute acid or sodium carbonate pretreatment on rice straw [40–42]. However, the reducing sugar yield from rice straw pretreated directly with B-8 was comparable to that from untreated rice straw.

Few of the above studies investigated bacterial pretreatment alone, or how the other prior pretreatments may affect a bacterial pretreatment. Therefore, in this study we investigated bacterial pretreatment by using B-6 to pretreat corn stover (CS) and propose a hypothesis to explain the low efficiency of enzymatic hydrolysis under the pretreatment by B-6 alone. Moreover, for the prior pretreatment method, we selected tetrahydrofuran–water (THF–H_2_O) co-solvent pretreatment to explain why the pre-erosion method may provide a beneficial effect for the pater bacteria pretreatment to further improve enzymatic hydrolysis. THF–H_2_O co-solvent is able to remove much of the lignin and hemicellulose. It is an effective chemical method for biomass deconstruction and delignification [43, 44].

## Methods

### Materials

CS was obtained from Shandong Province, China. THF (A.R. Shanghai Macklin Biochemical Co., Ltd) and concentrated sulfuric acid (98 wt%, ChengDu Chron Chemicals Co., Ltd) were purchased. The concentrated sulfuric acid was diluted to obtain the target concentration for each run. The cellulase (Cellic CTec2) was purchased from Novozymes. The original bacterial strain, B-6, had been deposited in the China General Microbiological Culture Collection Center with the accession number of CGMCC 4239. B-6 was activated and cultured in a Luria–Bertani medium. B-6 pretreatment of CS was applied in mineral salt medium (MSM, 2 g [NH_4_]_2_SO_4_, 1 g K_2_HPO_4_, 1 g KH_2_PO_4_, 0.2 g MgSO_4_, 0.1 g CaCl_2_, 0.05 g FeSO_4_, and 0.02 g MnSO_4_ in 1 L distilled water) [45]. All the chemicals of this MSM were purchased from a commercial source (Sinopharm Chemical Reagent Co., Ltd). Deionized water was used in all experiments reported here.

### B-6 pretreatment

The CS before pretreatment was milled to 18–60 mesh size and mixed with MSM at a volume ratio of 10:1 (g/l). This mixture was sterilized for 20 min at 121 °C. B-6 seed culture (20 mL) was activated overnight and then inoculated into 100 mL of Luria–Bertani broth medium in a rotary shaker at 30 °C with a speed of 150 rpm. When the activated B-6 seed solution had grown to optical density at 600 nm at 0.8–1.0, it was picked and centrifuged (at 6577×*g*, for 5 min) at a 10% volume ratio and the collected cells were inoculated into a sterile MSM containing CS sample at a volume ratio of 10:1 (g/L). B-6 pretreatment was carried out on a rotary shaker at 30 °C for 3 days at a speed of 150 rpm. After completing the pretreatment, the samples were filtered and washed with deionized water at least four times to remove any residual bacteria until the upper liquid was clear and then dried at 50 °C in an oven.

### Co-solvent pre-erosion before B-6 pretreatment

The milled CS of 18–60 mesh (20 g) was mixed with a THF–H_2_O solution (1:1, v/v) of 400 mL and 0.5 wt% H_2_SO_4_. Then, they were mixed and stirred with a glass rod for half a minute in a 500-mL hydrothermal reactor—stainless steel kettle body with a Teflon liner—before heating. Next, the reactor was heated to 150 °C in a blast-drying oven for 1, 1.5, 2, 4, and 6 h without stirring. After the pretreatment was complete, the reactor was quickly cooled by flushing with water. The solid sample and supernatant were separated through a 200-mesh screen. The solid samples were washed with deionized water many times to remove the THF, until the pH of the solution became neutral. Finally, the remaining residue was dried in an oven at 50 °C for 48 h. The following steps described below were likewise applied to the above B-6 alone pretreatment. In this study, the untreated CS, B-6 pretreated CS, THF–H_2_O pre-eroded CS, and THF–H_2_O pre-eroded combined with B-6 pretreated CS were named U-CS, B-CS, T-CS, and T-B-CS, respectively.

### Enzymatic saccharification and composition analysis

Batch enzymatic saccharification of the U-CS and pretreated CS samples was carried out at 50 °C in a water bath shaker. All samples were weighed in the ratio of 2.5% (w/v), with a total volume of 20 mL. The solution contained 20 mL of a citrate buffer (pH 4.8) with 30 μL of cellulase (Cllic CTec2, at 200 filter paper unit (FPU)/mL), 20 μL of tetracycline, and 20 μL of cycloheximide for the enzymatic hydrolysis. After 24-h incubation, 600 μL of the supernatant was taken and centrifuged at 6577×*g* for 5 min. Finally, we removed 100 μL of the supernatant to measure the total reducing sugars by the dinitrosalicylic (DNS) assay using glucose as the standard [46]. The composition of cellulose, hemicellulose, and lignin in the different samples was determined as described in a previous study [47, 48]. All measurements were carried out in batches, and experiments were performed in triplicate.

### Characterization methods

#### Morphology analysis

To observe the surface morphology change of the U-CS and the other pretreated CS groups, we selected representative samples for investigation under a scanning electron microscopy (SEM JSM-IT300LA) operated at 20 kV [49]. We also used an atomic force microscopy (AFM), and a NanoManTM VS + MultiMode V Scanning Probe Microscope (Veeco Company, USA) in tapping mode. Usually, three different areas per CS sample are investigated.

#### Structure analysis

The crystallinity index (CrI) of the U-CS and pretreated CS groups were analyzed by X-ray diffraction (XRD) (TTRIII, Rigaku Co., Tokyo, Japan) [50]. Samples were scanned at a speed of 2° min^−1^ from 10° to 30°. The step size was set at 0.01°. CrI was calculated according to the following equation [51]:

$$ {\text{CrI = (}}I_{002} - I_{\text{am}} )/I_{ 0 0 2} \times 100\% , $$

where *I*_002_ is the peak intensity corresponding to the (002) lattice plane of cellulose I, and *I*_am_ is the peak intensity observed at 2θ = ~ 18.5°. Fourier Transform infrared (FTIR) spectra of the CS samples were recorded on a Nicolet IS10 spectrometer in the range 4000–400 cm^−1^ [52]. The CS samples were mixed with KBr, ground to a fine powder, and pressed into pellets for the infrared transmission studies. FTIR was used to determine the characteristic absorption peaks of the chemical bonds in CS and the isolated lignin from CS [53].

#### HSQC-2DNMR analysis

All hetero-nuclear single quantum coherence (HSQC) nuclear magnetic resonance (NMR) experiments were acquired in a Bruker Avance 400 MHz spectrometer with a 5 mm Broadband Observe probe. The isolation of lignin was performed according to a previous study [53]. Lignin from the CS samples was added to 0.5 mL of dimethyl sulfoxide (DMSO)-d_6_. A standard Bruker HSQC pulse sequence (HSQCETGP) was applied under the following conditions: 13 ppm spectral width in the F2 (^1^H) dimension with 1024 data points, 210 ppm spectral width in the F1 (^13^C) dimension with 256 data points, a 1.5 s pulse delay, a 90° pulse, and a *J*_*C*−*H*_ of 145 Hz and 128 scans [54].

#### Porous analysis

Nitrogen porosimetry (Micromeritics ASAP 2460) was used to measure the specific surface area (SSA) and pore volume (PV) of the U-CS and pretreated CS groups. The SSA and PV of CS samples were estimated by the Brunauer–Emmett–Teller (BET) method with nitrogen gas adsorption at 77.3 K [55]. Before this determination, the samples were first dried for 3 h in vacuum at 150 °C. Subsequently, the samples were degassed at 150 °C for 4 h in the degassing system to remove the water and impurities and cooled in the presence of nitrogen gas. The obtained samples were weighed by the scale in the instrument. The tests were automatically started until the mass of samples reached a balance. The analysis condition was as follows: sample mass, about 0.45 g; the equilibration interval, 10 s; warm free space, 15.9191 cm^3^; cold free space, 46.9843 cm^3^; and sample density: 1.000 g/cm^3^.

#### Surface structure analysis

The surface elemental composition and the surface lignin coverage (SLC) of CS were determined by X-ray photoelectron spectroscopy (XPS, Thermo 2500XI) [56]. SLC was calculated from the O/C ratios according to the following equation:

$$ {\text{SLC}} = ({\text{O/C}}_{\text{sample}} - {\text{O/C}}_{\text{lignin}} - {\text{O/C}}_{\text{cellulose}} ), $$

where the O/C_sample_ term is the O/C ratio of the analyzed sample, and O/C_cellulose_ and O/C_lignin_ are the theoretical O/C ratios of pure cellulose (0.83) and lignin (0.33) [12, 57].

### Statistical analysis

All samples were tested in triplicate. Error bars showing in relative figures represent standard deviations of triplicate samples. The number of samples used for the analysis is 3. The experimental results were validated by statistical analysis using Origin 8.5 software.

## Results and discussion

### B-6 pretreatment

**Fig. 1:**
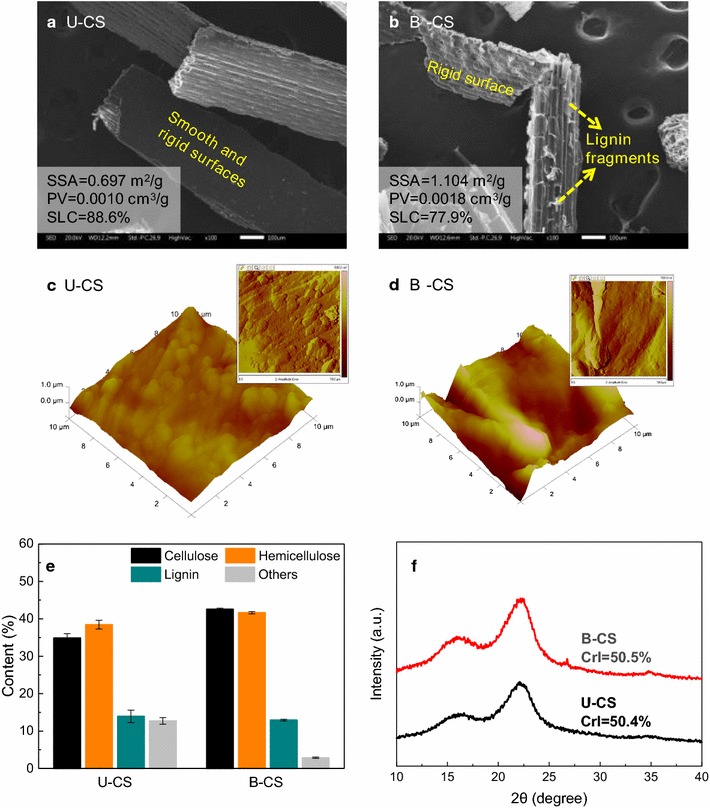
Morphology and composition of U-CS and B-CS. **a** SEM image of U-CS, **b** SEM image of B-CS, **c** AFM images of U-CS, **d** AFM images of B-CS, **e** composition of U-CS and B-CS, and **f** XRD patterns of U-CS and B-CS. Error bars shown in (**e**) are standard deviations of triplicate samples

We first performed the application of B-6 in CS pretreatment. Both the U-CS and B-CS groups were analyzed by the SEM, AFM, and BET assays, as shown in Fig. 1. According to Fig. 1a, c, U-CS has a smooth and rigid surface structure, while its SSA and PV were 0.697 m^2^/g and 0.0010 cm^3^/g, respectively (the data of SSA and PV are shown in Additional file 1: Figure S2). These structural characteristics of U-CS were attributed to the high lignin coverage on its surface, as evidenced by a high SLC value of 88.6% (from the XPS analysis). After B-6 pretreatment, the outer structure of B-CS appeared as sheet-like pleats; this may indicate lignin fragmentation by B-6. Accordingly, the SSA and PV of B-CS increased to 1.104 m^2^/g and 0.0018 cm^3^/g, respectively. However, this porosity was still lower than 1.33 m^2^/g and 0.004 cm^3^/g of the untreated rice straw [58], and thus insufficiently high to contribute to a high enzymatic digestibility. Similarly, though B-6 pretreatment slightly decreased the SLC of the B-CS group to 77.9%, this value was still higher than 65.5% of the readily digestible biomass [59]. Notably, the dense and continuous surfaces remained distinct as observed under the SEM (Fig. 1b) and AFM (Fig. 1d) images of B-CS, thus indicating the retained integrity of the B-CS microstructure, despite the partial fragmentation of lignin by B-6.

The composition of U-CS and B-CS groups was analyzed (Fig. 1e). The U-CS group was found to consist of 34.9% cellulose, 38.5% hemicellulose, and 13.9% lignin, along with 12.7% of other organic and non-organic components, such as ash, proteins, lipids, and extractives [20]. However, after B-6 pretreatment, the content of this other matter was reduced to 2.9%. The cellulose and hemicellulose contents slightly increased to 42.6% and 41.6%, respectively. However, B-6 pretreatment caused an insignificant reduction in the lignin content of B-CS (12.9%). In addition, comparing the XRD patterns of U-CS and B-CS revealed that their crystallinity was similar (CrI, 50.4–50.5%). The composition and XRD results thus confirmed that large amounts of amorphous lignin were retained on the B-CS surface.

**Fig. 2:**
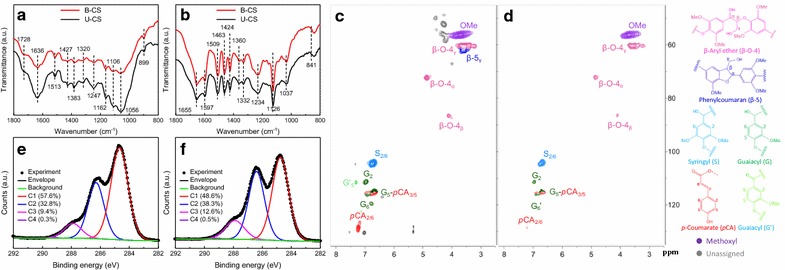
Chemical structure of U-CS and B-CS. **a** FTIR spectra of U-CS and B-CS, **b** FTIR spectra of lignin extracted from U-CS and B-CS, **c** HSQC spectrum of lignin extracted from U-CS, **d** HSQC spectrum of lignin extracted from B-CS, **e** XPS results for U-CS and, **f** XPS results for B-CS

The FTIR analysis investigated possible changes in the chemical structure of U-CS and B-CS (Fig. 2a). Additional file 1: Table S1 lists the assignments of the major FTIR peaks we found. Evidently, the FTIR characterizations revealed no discernible difference between the U-CS and B-CS groups, indicating that B-6 pretreatment did not alter the chemical structure of the U-CS. This result was unexpected because B-6 has been recognized as effective lignin-degrading bacteria [36]. For further confirmation, the lignin in both U-CS and B-CS was extracted and characterized by the FTIR and HSQC analysis (Fig. 2b–d). The HSQC spectra showed that the signal for *β*-5_γ_ disappeared and the signals for the G-type lignin became weaker in B-CS compared to U-CS, possibly due to lignin fragmentation by B-6 pretreatment, in line with the SEM observations. However, the FTIR spectra showed that the chemical structure of lignin before and after B-6 pretreatment was consistent (Fig. 2b). Based on these HSQC and FTIR results, it was difficult to reach a satisfactory conclusion, possibly because B-6 impacts on lignin only occurred at the CS surface.

Herein, to obtain greater understanding, a surface-selective analytical technique (XPS) was performed on the U-CS and B-CS groups. Deconvoluting the XPS C_1s_ peak should help to identify the changes in prevalent carbon species on the CS surface [60]. Evidently, the dominant peak at 284.8 ± 0.1 eV (C1) corresponded to non-functionalized carbon atoms located in aromatic rings and in the aliphatic side chains generally from lignin. The peak at 286.4 ± 0.1 eV (C2) was ascribed to the carbon atoms bonding to single non-carbonyl oxygen molecules (C–O). In addition, the peaks at 287.9 eV (C3) and 289.3 eV (C4) were associated with the carbon atoms bonding to a carbonyl or two non-carbonyls (C=O or O–C–O) and the carbon atoms bound in esters or carbonic acids (O–C=O) [60], respectively. Unlike the FTIR results, the XPS analysis showed a clear transformation of the carbon species on the CS surface after B-6 pretreatment. Specifically, there was a reduction of C–C bonds (C1), from 57.6 to 48.6%, along with an increase in the abundances of both C–O bonds (C2) and C=O/O–C–O bonds (C3), possibly resulting from a lignin depolymerization that occurred on the CS surface. Notably, the C–C bond was still the prevalent carbon species on the B-CS surface, which suggested that lignin was largely retained after B-6 pretreatment.

**Fig. 3:**
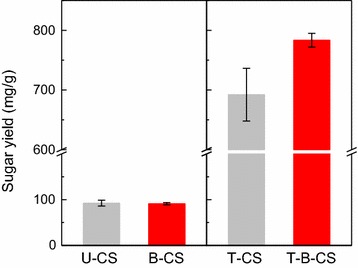
Enzymatic digestibility (sugar yield) of U-CS, B-CS, T-CS, and T-B-CS. Error bars shown are standard deviations of triplicate samples

Taken together, these results demonstrate that the function of B-6 pretreatment upon lignin was to slightly cause its fragmentation, but only at biomass surface without exposing the internal cellulose. As a result, there was no obvious increase in the enzymatic digestibility, i.e., the sugar yield of U-CS was 84.7 mg/g, while that of B-CS was 92.1 mg/g (Fig. 3). This undesirable effect of the bacterial pretreatment was related to the enzymatic mechanism, but this is still unclear. Generally, it is accepted that fungi could decompose lignin by using their ligninolytic enzymes, including LiP, MnP, VP, and Lac [61, 62]. Indeed, our previous study found a relatively high activity of Lac and MnP for B-6 when it was cultured in a medium with Kraft lignin as sole carbon source [36]. For this reason, Lac and MnP were expected to play an important role in the effects of B-6 pretreatment. However, these ligninolytic enzymes, per se, are not thought to react directly with lignin in the fungal system [63]. Instead, in the current working model, the oxidation of lignin is related to a broad range of small molecule oxidants acting as diffusible mediators, which directly react with lignin to generate both phenoxyl and phenyl radicals on the substrate that initiates a cascade of bond scission reactions [64]. It is acknowledged, however, that bacteria may exhibit different reactivities toward lignin than fungi, i.e., the bacterial system may not be completely mediated via small molecule oxidants [65]. There are some direct interactions that occur between the enzymes and lignin substrates in the bacterial system [63]. This may well be the main reason for the low efficiency of the bacterial system in the biomass pretreatment found here. Thus, the existence and diffusion of mediators could be essential conditions for lignin damage to proceed in a biological pretreatment. However, based on the above results, no such mediators were detected. More seriously, the highly dense and compact surface architecture of U-CS would inhibit the binding and transportation of any mediators. To address this point, a pre-erosion operation on biomass surface may be required for an effective biological pretreatment. Pre-erosion (or pre-modification) of biomass by an ultrasound or H_2_O_2_ treatment supposedly increased the accessibility of fungal enzymes [57, 66], but this view lacks substantial evidence. In the current study, we adopted a co-solvent method that used the THF–H_2_O system to pre-erode the CS surface to verify the assumption that the THF–H_2_O system attacks the lignin to expose more lignin substrates, which are used as mediators for Lac and MnP on lignin depolymerization during B-6 pretreatment, and to provide further evidence for exploring the involved pre-erosion mechanism.

### Pre-erosion enhances B-6 pretreatment

**Fig. 4:**
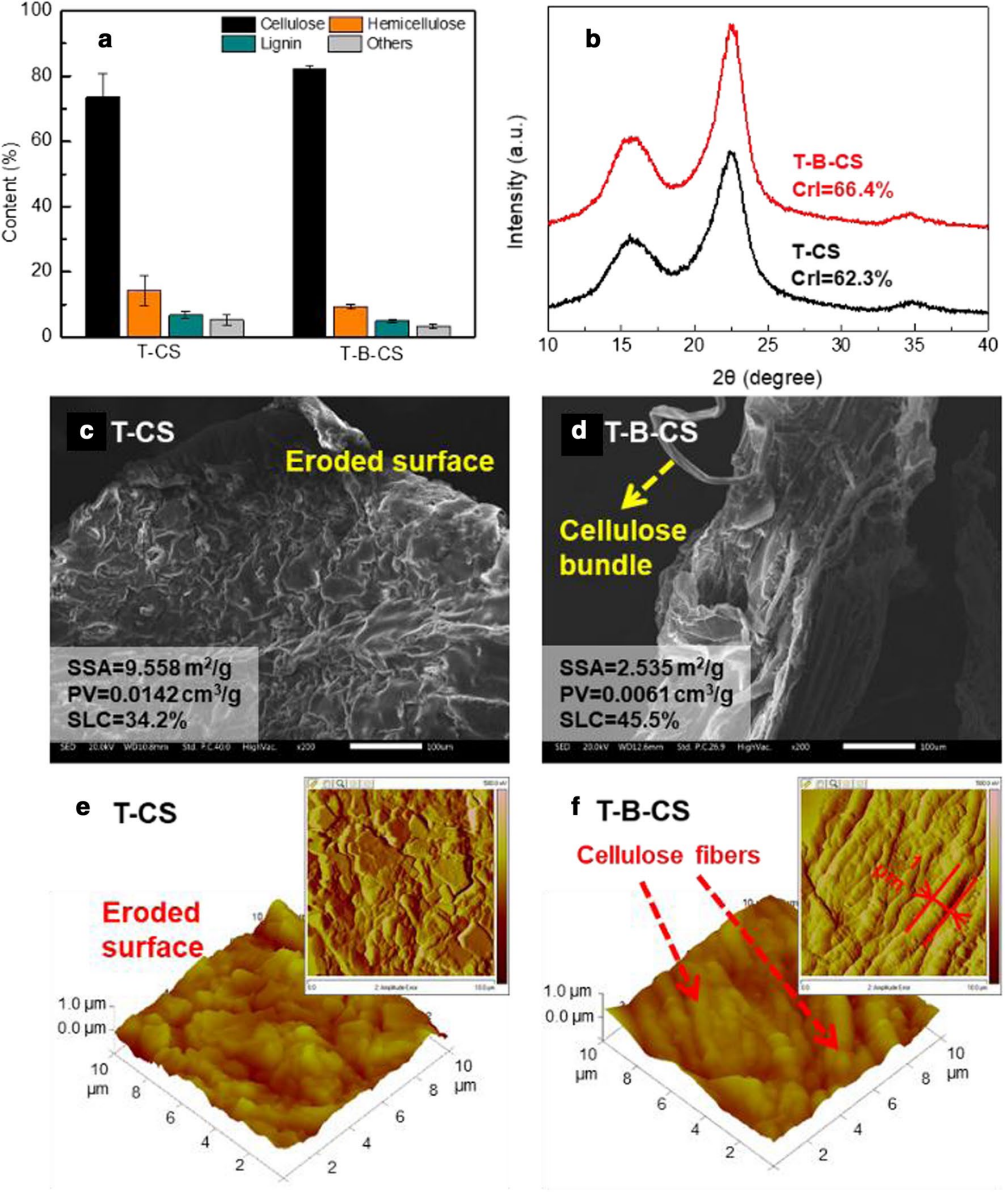
Composition and morphology of T-CS and T-B-CS. **a** Composition of T-CS and T-B-CS, **b** XRD patterns of T-CS and T-B-CS, **c** SEM image of T-CS, **d** SEM image of T-B-CS, **e** AFM images of T-CS, and **f** AFM images of T-B-CS. Error bars shown in (**a**) are standard deviations of triplicate samples

The literature [12, 67] has reported that an acid-assisted co-solvent system could produce a desirable result when used under a relatively low temperature. Therefore, in this work, we employed an H_2_SO_4_-assisted THF–H_2_O system to pre-erode U-CS, with the pre-erosion time optimized to 4 h to maximize the effect (Additional file 1: Figure S1). In fact, the THF–H_2_O system has already been identified as a versatile co-solvent for both lignin removal and cellulose retention, thus enabling it to function as an efficient biomass pretreatment method on its own [68]. As Fig. 4a shows, the contents of hemicellulose, lignin, and other matter in T-CS were reduced to 14.4, 6.7, and 5.3%, respectively. Accordingly, there was a significant enrichment in the cellulose content (73.6%) in the T-CS group compared to the U-CS and B-CS in Fig. 1e. The XRD data (Fig. 4b) showed a marked increase in the crystallinity for T-CS (CrI, 62.3%) when compared with the U-CS. This was not surprising since the THF–H_2_O system removed a substantial amount of amorphous hemicellulose and lignin; hence, crystalline cellulose dominated. Such a high content of cellulose contributed to a 6.5-fold increase in the enzymatic digestibility of T-CS over U-CS (Fig. 3).

More significantly, the results in Fig. 3 show that T-B-CS exhibited a sugar yield that was 91.2 mg/g greater than that of T-CS, and 7.5-fold higher than that of U-CS. In addition, that cellulose content was enriched to 82.4% in T-B-CS, because B-6 pretreatment further removed the noncellulosic fractions. The lignin content in T-B-CS was reduced by 26% when compared with T-CS. Similar phenomena were also observed for the other T-B-CS samples treated under various pre-erosion conditions (Additional file 1: Figure S1). These results, in a preliminary way, verified our assumption that the THF–H_2_O pretreatment favored B-6 pretreatment. To track the mechanism involved, however, we subsequently evaluated the morphology and chemical structure of both T-CS and T-B-CS.

The SEM and AFM images of T-CS and T-B-CS are presented in Fig. 4. Quite unlike either U-CS or B-US, the surface of T-CS had changed to become very rugged with a remarkable increase in porosity: the SSA increased to 9.558 m^2^/g and PV increased to 0.0142 cm^3^/g. Such high porosity allowed sufficient binding of the bacterial enzymes to T-CS, as well as the easy movement of mediators inside [69]. In addition, since much lignin was removed—as demonstrated by composition analysis—the SLC significantly decreased to 34.2% for T-CS (Fig. 4c). However, it was difficult to find the cellulose bundle structure in either the SEM or AFM views of T-CS, indicating that the inner cellulose was still encased by residual lignin. However, after B-6 pretreatment, the situation had changed. Although the SLC of T-B-CS increased slightly to 45.5%, possibly due to the redistribution of lignin by B-6, the individual cellulose bundle was now loosened and segregated from the bulk matrix. The AFM observations highlighted the differences in the surface architecture, showing a regularly banded surface for the T-B-CS group: this indicates that the cellulose bundle observed in the SEM images was an aggregate of cellulose fibers with a width of ~ 1 μm. It is also worth noting that the porosity of T-B-CS was decreased, while the values for SSA and PV were reduced to 2.535 m^2^/g and 0.0061 cm^3^/g, respectively. This result agrees with the findings reported in several related studies [70] and suggests that the bulky removal of hemicellulose and lignin leads to pore collapse.

**Fig. 5:**
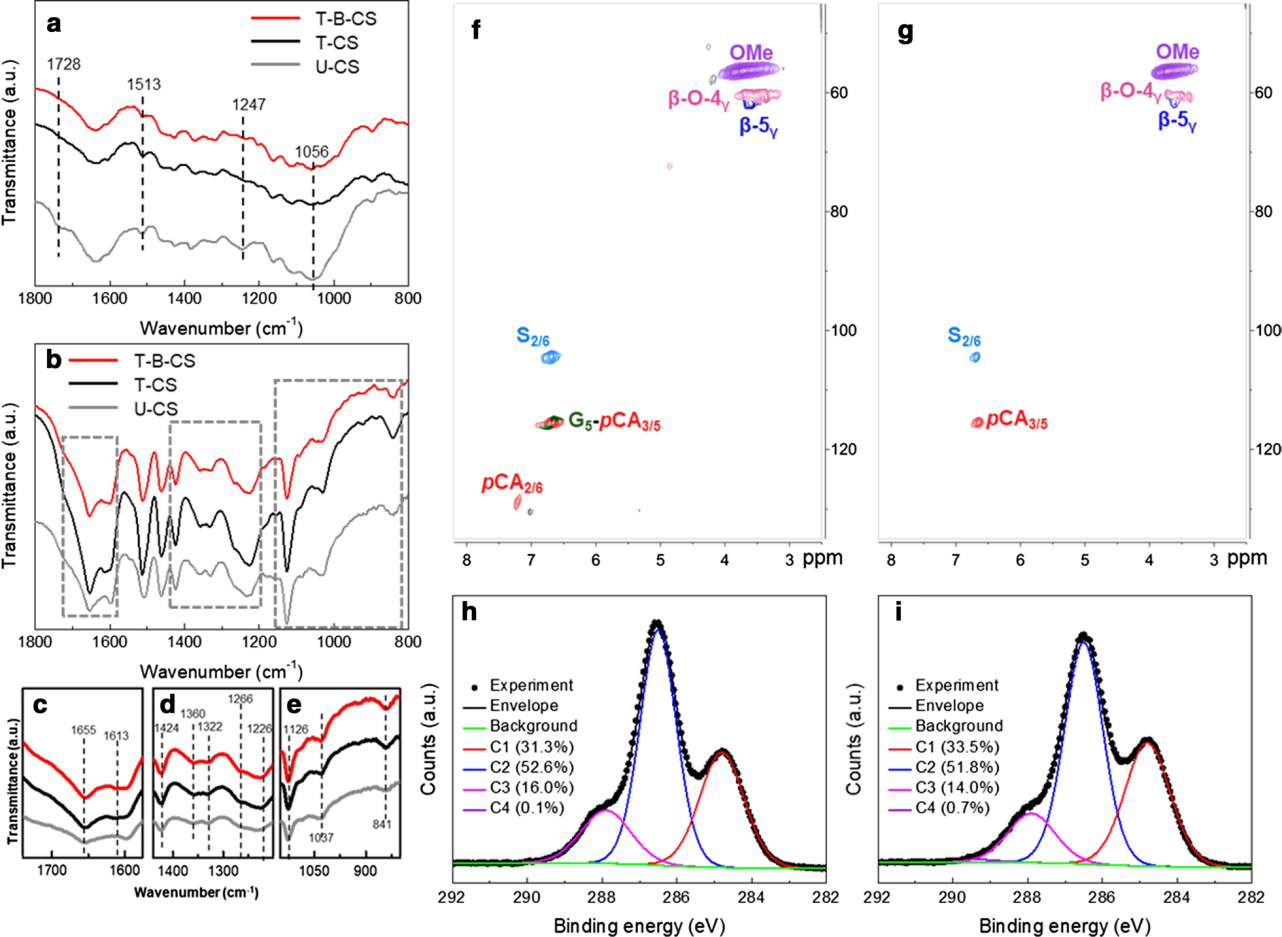
Chemical structure of T-CS and T-B-CS. **a** FTIR spectra of T-CS, T-B-CS and U-CS, **b**–**e** FTIR spectra of lignin extracted from T-CS, T-B-CS and U-CS, **f** HSQC spectrum of lignin extracted from T-CS, **g** HSQC spectrum of lignin extracted from T-B-CS, **h** XPS results for T-CS, and **i** XPS results for T-B-CS

The molecular mechanism of pre-erosion was investigated by the FTIR, HSQC, and XPS analyses. From the obtained FTIR spectra (Fig. 5a), the band at 1728 cm^−1^ disappeared in the T-CS group compared with U-CS, indicating the removal of noncellulosic polysaccharides by the THF–H_2_O system [71]. In addition, compared to U-CS, the bands typical for the stretching of C–O in hemicellulose (1247 and 1056 cm^−1^) were weakened and deformed, thus giving additional evidence for the hemicellulose removal [72]. The FTIR spectrum of lignin extracted from T-CS (black line in Fig. 5b–e) clearly differed from that of U-CS (gray line in Fig. 5b–e). Specifically, pre-erosion contributed to a smoother peak visible at 1332 cm^−1^, indicating the structural modification of S-type derivatives by the THF–H_2_O system, but with no obvious degradation of the syringyl ring (i.e., no change in the peaks at 1424, 1126, and 841 cm^−1^). The peaks around 1508 and 1600 cm^−1^ will change if the C=C stretches from aromatic rings or aromatic skeleton vibrates. Furthermore, the peak attributed to the stretching of C=O conjugated to aromatic rings (1655 cm^−1^) became sharper. A new peak assigned to the C=O stretching at 1613 cm^−1^ had appeared. The peak attributed to a ring breathing motion with C–O stretching now shifted from 1234 to 1226 cm^−1^, corresponding to ring breathing with C=O stretching. These carbon species-related changes perhaps indicated that some linkages (such as *β*-O-4, *β*-5) in lignin were broken, thereby exposing the C–O and C=O bonds [73].

This was confirmed by the HSQC spectrum of lignin extracted from T-CS (Fig. 5f). Compared to U-CS (Fig. 2c), clearly, the signals of *β*-O-4 and *β*-*5* linkages were weakened. In addition, the intensity of S- and G-type lignins decreased, suggesting the breakdown of lignin by the THF–H_2_O system. Further evidence for the changes to the carbon species on the CS surface from pre-erosion was obtained from XPS analysis. As Fig. 5h shows, the abundance of C1 on the T-CS surface clearly decreased to 31.3%. By contrast, the strength of the C2 peak increased significantly for T-CS, reaching an abundance of 52.6%, while the abundance of C3 increased slightly, to 16.0%, demonstrating that C–O and C=O species were exposed on the T-CS surface. Considering the FTIR, HSQC and XPS results together, it could be concluded that pre-erosion via the THF–H_2_O system cleaved the *β*-O-4 and *β*-*5* linkages in lignin without degrading the benzene ring, which likely led to some phenoxy radicals becoming exposed [74, 75]. These lignin derivatives were then re-deposited on the T-CS surface due to their low solubility under acid conditions, thus contributing to the formation of a rough surface.

**Fig. 6:**
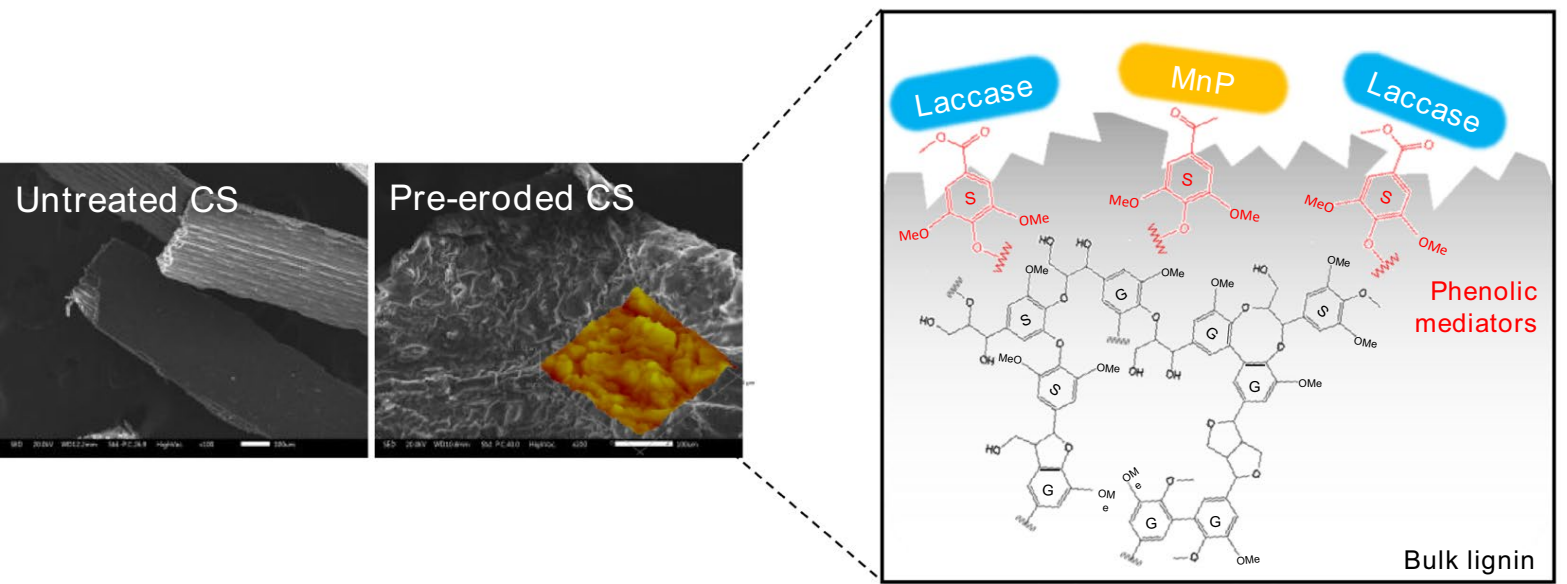
The proposed mechanism graph of how pre-erosion enhances B-6 pretreatment

As mentioned above, the bacterial system applied in this study could not be mediated by small molecule oxidants which are embedded in lignin networks. However, the effect of Lac on biomass pretreatment is reportedly enhanced by some mediators, such as the veratryl alcohol cation radical and various Mn(III) coordination complexes or those produced in secondary radical cascades [63]. Recently, it was also found that phenoxy radicals generated from syringyl phenolics could act as diffusible mediators in the lignin modification via Lac or MnP [69]. To our surprise, the result of this study suggested the re-deposition of some phenoxyl radicals on the T-CS surface. Moreover, we also found that irrespective of Lac or MnP activity, B-CS maintained a higher activity than T-B-CS (Additional file 1: Figure S2); this result was similar to that of a previous report [20], which may be explained by the more number of phenoxy radicals and the lower enzymes (MnP and Lac) needed to achieve the depolymerization of lignin. Therefore, these phenoxy radicals could have an important role to play as the mediators in the ligninolytic enzyme system in bacterial pretreatment, since the rough and porous surface supports their transportation and diffusion (Fig. 6). Correspondingly, this pre-erosion action made B-6 pretreatment easy and highly efficient. As evidenced by the FTIR spectrum of lignin extracted from T-B-CS, the shoulder peak at 1266–1270 cm^−1^ was sharper, indicating a structural change of the G-type lignin by B-6 pretreatment. In addition, the characteristic peak of the G-type (1037 cm^−1^) [76] became weaker and demonstrated that it was the main target of B-6 pretreatment. Similarly, the HSQC results also showed that the signals of G-type lignin disappeared in the T-B-CS group. The contribution of different types of carbon on T-B-CS showed no obvious changes when compared with T-CS as shown in Fig. 5h, i, possibly due to the significant removal of lignin on the surfaces of both T-CS and T-B-CS. In summary, we conclude that pre-erosion eases and fosters the action of B-6 pretreatment by providing phenoxy radicals as mediators and building a rough surface architecture, eventually contributing to a higher enzymatic digestibility of T-B-CS.

## Conclusions

The enzymatic digestibility of B-CS showed no increase when compared with U-CS. Based on the morphology and structure characterization for U-CS and B-CS, the bacteria B-6 caused lignin fragmentation but only damaged the structure on the CS surface, possibly due to insufficient mediators and attachment sites on the CS surface for ligninolytic enzymes of B-6. In addressing this point, we used the acid-catalyzed THF–H_2_O system to pre-erode CS. Those results showed that pre-erosion could remove the lignin and hemicellulose biomass substantially. More importantly, pre-erosion promoted the formation of lignin derivatives, i.e., phenoxy radicals, to mediate the interactions between the ligninolytic enzymes and lignin. Furthermore, these lignin derivatives could then be re-deposited on the CS surface, contributing to a rough and porous architecture that eases the diffusion and transport of key mediators. The results of this study provided strong empirical evidence that mediators play important role in bacterial or enzymatic pretreatment and offer guidance for the design of new biological pretreatment for the lignocellulose biorefinery process.

## Additional file

**Additional file 1: Table S1.** Main assignments of FTIR bands of CS. **Figure S1.** Enzymatic digestibility of CS pretreated under different conditions. **Figure S2.** SSA and PV of different treated CS samples. **Figure S3.** Lac and MnP activity of B-CS and T-B-CS.

## Abbreviations

CS: corn stover
B-6: *Pandoraea* sp. B-6
THF–H_2_O: tetrahydrofuran–water
MnP: manganese peroxidase
Lac: laccase
LiP: lignin peroxidase
VP: versatile peroxidase
MSM: mineral salt medium
CrI: crystallinity index
SLC: surface lignin coverage
U-CS: untreated corn stover
B-CS: B-6 pretreated corn stover
T-CS: THF–H_2_O pretreated corn stover
T-B-CS: combined THF–H_2_O with B-6 pretreated corn stover
SSA: specific surface area
PV: pore volume
