# Supplementary material for: Use of bacteria for improving the lignocellulose biorefinery process: importance of pre-erosion
Source: Biotechnol Biofuels. 2018 May 23;11:146. doi: 10.1186/s13068-018-1146-4 (PMC5964970; doi:10.1186/s13068-018-1146-4)
Supplement: Supplementary file 1 — Additional file 1: Table S1. Main assignments of FTIR bands of CS. Figure S1. Enzymatic digestibility of CS pretreated under different conditions. Figure S2. SSA and PV of different treated CS samples. Figure S3. Lac and MnP activity of B-CS and T-B-CS. [file 13068_2018_1146_MOESM1_ESM.docx]

# Supporting Information

# Use of bacteria for improving the lignocellulose biorefinery process: Importance of Pre-erosion

*Shengnan Zhuo ^1,&^, Xu Yan* *^1, 2,&^ , Dan Liu ^1^, Mengying Si ^1^, Kejing Zhang ^1^, Mingren Liu* *^1^, Bing Peng ^1,2^,*

*Yan Shi ^1, 2, *^*

^1^ School of Metallurgy and Environment, Central South University, Changsha 410083, China

^2^ Chinese National Engineering Research Center for Control & Treatment of Heavy Metal Pollution, Changsha 410083, China

^&^ These authors contributed equally to this work.

*^*^* Corresponding author: [shiyzyrs@csu.edu.cn](mailto:shiyzyrs@csu.edu.cn)

**Table S1 Main assignments of FTIR bands of CS[**[**1-3**](#_ENREF_1)**]**

| Wavenumbers (cm^-1^) | Assignments |
| --- | --- |
| 1728 | C=O stretching from esters, waxes, fatty acids, and noncellulosic polysaccharides |
| 1655 | Stretching of C=O conjugated to aromatic rings |
| 1632-1636 | Bending caused by absorbed water in cellulose |
| 1613 | Carbonyl group stretching |
| 1594-1609 | Aromatic ring vibrations |
| 1504-1515 | -CH bending in the aromatic rings of lignin |
| 1462-1464 | Asymmetric C-H bending (in CH_3_ and -CH_2_-) in lignin |
| 1427 | CH_2_ bending in cellulose |
| 1421-1424 | Aromatic ring vibrations |
| 1383 | Symmetrical stretching for C=O of COO- groups in |
| 1360 | Aliphatic C-H stretch in the methyl group (not MeO) and the phenolic OH |
| 1320 | C-H in cellulose |
| 1332 | C=O stretching in syringyl derivatives |
| 1266-1270 (shoulder) | Guaiacyl ring stretching vibrations |
| 1247 | Acetate ester C-O stretching from hemicellulose |
| 1234 | Ring breathing with C-O stretching |
| 1226 | Syringyl and guaiacyl ring breathing with C=O stretching |
| 1160-1162 | Asymmetric C-O-C bridge stretching in cellulose |
| 1126 | Aromatic skeletal and C-O stretch |
| 1106-1112 | Anhydroglucose ring in cellulose |
| 1056 | C-O stretch in cellulose and hemicellulose |
| 1037 | Aromatic C-H in-plain deformation for guaiacyl type |
| 899 | C-O-C stretch from glycosidic linkage between glucose units of cellulose |
| 841 | Aromatic C-H out of plane bending |

The Table S1 shows the main assignments of FTIR bands of CS, which helps to understand the changes in the chemical structure of CS.

**Reference:**

1. Filipe Natalio RF, Sidney R. Cohen, Gregory Leitus, Gerhard Fritz-Popovski, Oskar Paris, Michael Kappl, Hans-Jürgen Butt. Biological fabrication of cellulose fibers with tailored properties. Science. 2017;357:1118-1122.

2. Tripathi A, Ferrer A, Khan SA, Rojas OJ. Morphological and Thermochemical Changes upon Autohydrolysis and Microemulsion Treatments of Coir and Empty Fruit Bunch Residual Biomass to Isolate Lignin-Rich Micro- and Nanofibrillar Cellulose. ACS Sustain Chem Eng. 2017; 5:2483-2492.

3. Rangan A, Manchiganti MV, Thilaividankan RM, Kestur SG, Menon R. Novel method for the preparation of lignin-rich nanoparticles from lignocellulosic fibers. Ind Crops Prod. 2017;103:152-160.

**Figure S1 Enzymatic digestibility of CS pretreated under different conditions. Error bars shown are standard deviations of triplicate samples.**

Figure S1 shows the reducing sugar yield of CS pretreated under different conditions, obviously, the enzymatic digestibility was the best under the condition of 150 ^o^C, 4 h. As the same, the highest sugar yield occured by B-6 treatement after this condition. So, in the text, we selected a group of samples under this condition as the representative to study.

**
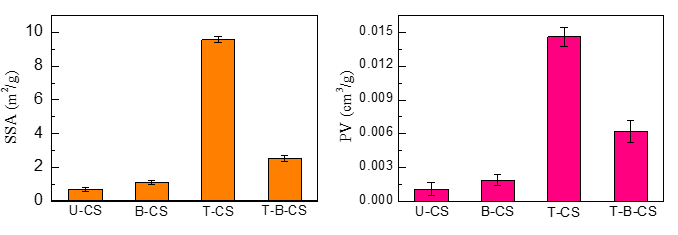
**

**Figure S2 SSA and PV of CS samples. Error bars shown are standard deviations of triplicate samples.**

Figure S2 shows the SSA and PV of the representative CS samples. Due to the destruction of CS by THF–H_2_O co-solvent system, the SSA and PV of T-CS was much higher than U-CS. The hole collapse of T-B-CS resulted in the decrease of SSA and PV, which was attributed to the further removal of lignin after the modification by B-6. But the enzymatic hydrolysis is still improved.


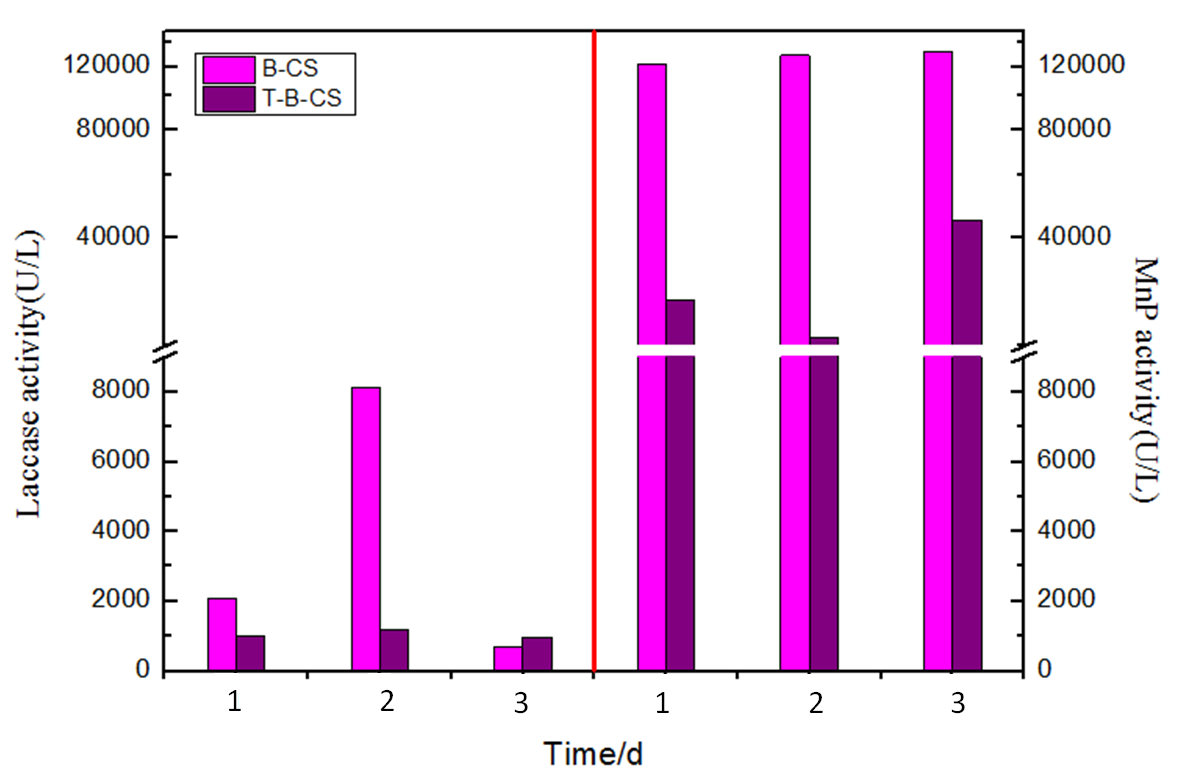


**Figure S3 Lac and MnP activity of B-CS and T-B-CS**

Figure S3 is a reflection of enzyme activity including MnP and Lac. All the data suggested the activity of B-CS was higher than the T-B-CS, indicating the enzymes producing by B-6 were consumed by the releasing phenols from the T-CS.
